# Supplementary material for: CRUX, a platform for visualising, exploring and analysing cancer genome cohort data
Source: NAR Genom Bioinform. 2024 Feb 1;6(1):lqae003. doi: 10.1093/nargab/lqae003 (PMC10833466; doi:10.1093/nargab/lqae003)
Supplement: lqae003_Supplemental_File [file lqae003_supplemental_file.pdf]

SUPPLEMENTARY TABLES

Property	TCGA Biolinks GUI	cBio Portal	St. Jude Cloud	Gene Pattern	CVCDAP	Maf tools	CRUX
**Inbuilt support for public data analysis**	✓	✓	✓	✓	✓	✓	✓
**Supports analysis of unpublished user data**	✓	✓ (orange)	✓ (orange)	✓	✓	✓	✓
**Runs locally (Windows/ MAC)**	✓					✓	✓
**Dedicated inter-operability module**							✓

Supplementary Table S1. Key features of cancer cohort-level analysis suites. Green tick symbol: key features supported. Orange tick symbol: functionality is technically supported but with at least one of the following caveats: 1) technically demanding creation of a local instance, 2) resource maintainers need to be contacted to organise integration of user data, 3) upload of data to paid cloud services, or 4) code needs to be written or run by end-users.

Class	Type of Variation	Analysis / Visualisation
Single Cohort Analyses	SNVs and Indels	Oncoplot
	SNVs and Indels	TiTv Rates
	SNVs and Indels	Highly Mutated Pathways
	SNVs and Indels	Gene level lollipop plots
	SNVs and Indels	Pfam Domain Mutation rates
	SNVs and Indels	Somatic Interactions (gene-level cooccurrence of mutations)
	SNVs and Indels	Survival Analysis for prognostic biomarker identification
Enrichment Analysis	SNVs and Indels	Genomic Enrichment Analysis
Two-Cohort Analysis	SNVs and Indels	Cohort Comparison
Single Cohort	CNVs	GISTIC2 Chromplot
		GISTIC2 Oncoplot
Sample Level	SNVs	Kataegis
Sample Level	Heterogeneity	Heterogeneity estimation
Utilities	SNVs and Indels	Subset / Merge Cohorts

Supplementary Table S2: Modules available in CRUX

SUPPLEMENTARY FIGURES

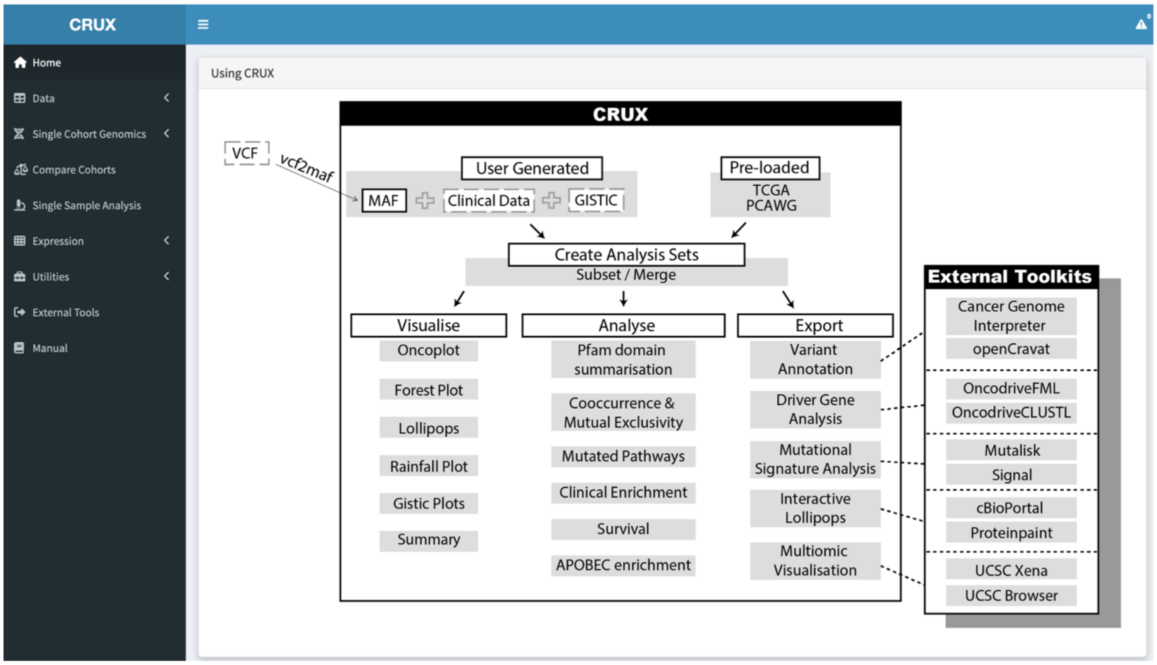

Supplementary Figure 1. CRUX landing page. This screenshot of the CRUX user interface window shows the available workflows, the organisation of tools into visualisation, analysis and export tools for using several external toolkits.

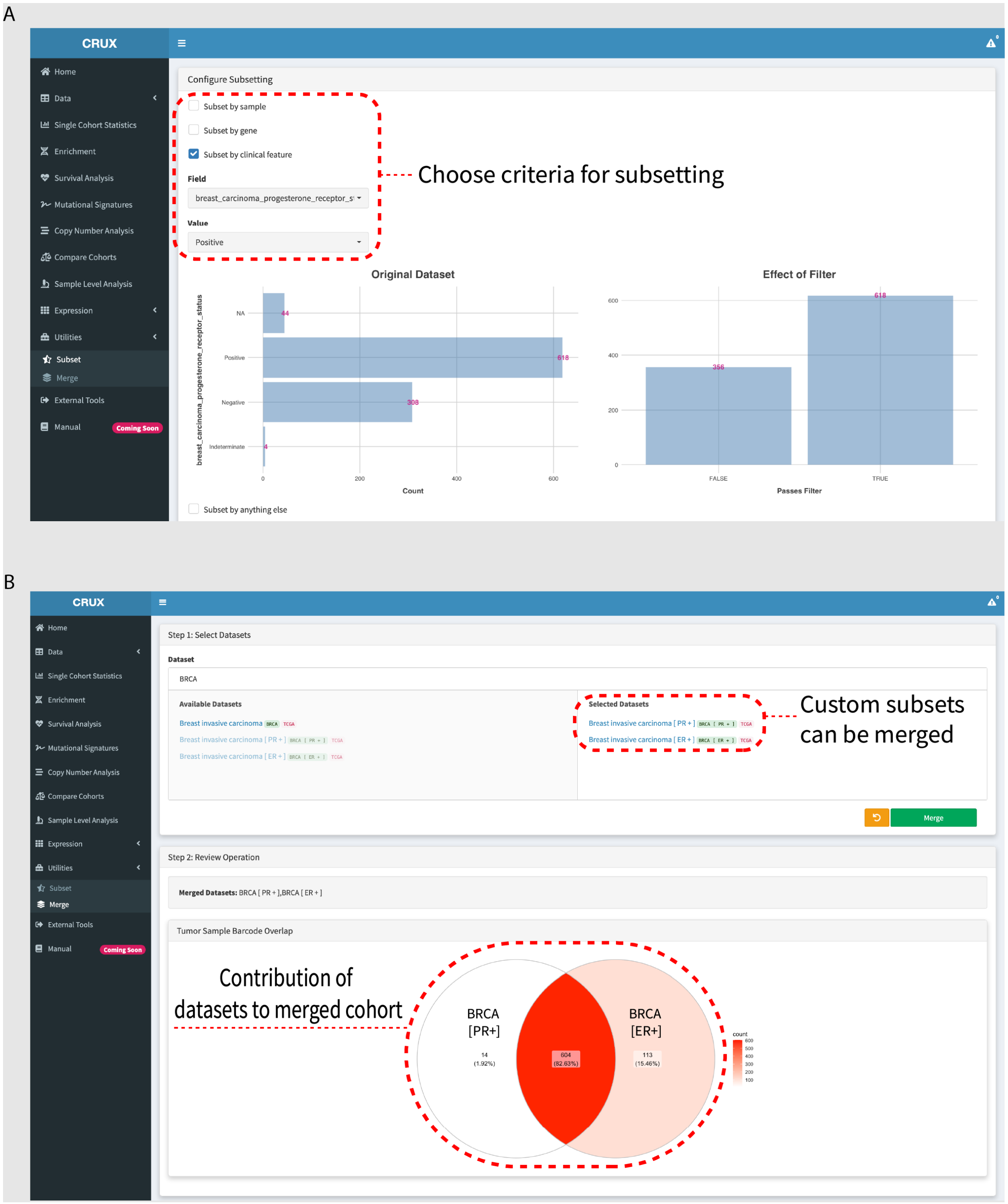

Supplementary Figure 2. **Creation of new cohorts in CRUX**. This is illustrated by screenshots of CRUX user interface window. (A) Subsetting and (B) merging of cohorts to create new analysis sets. Subsetting can be based on molecular abberations or sample-level metadata.

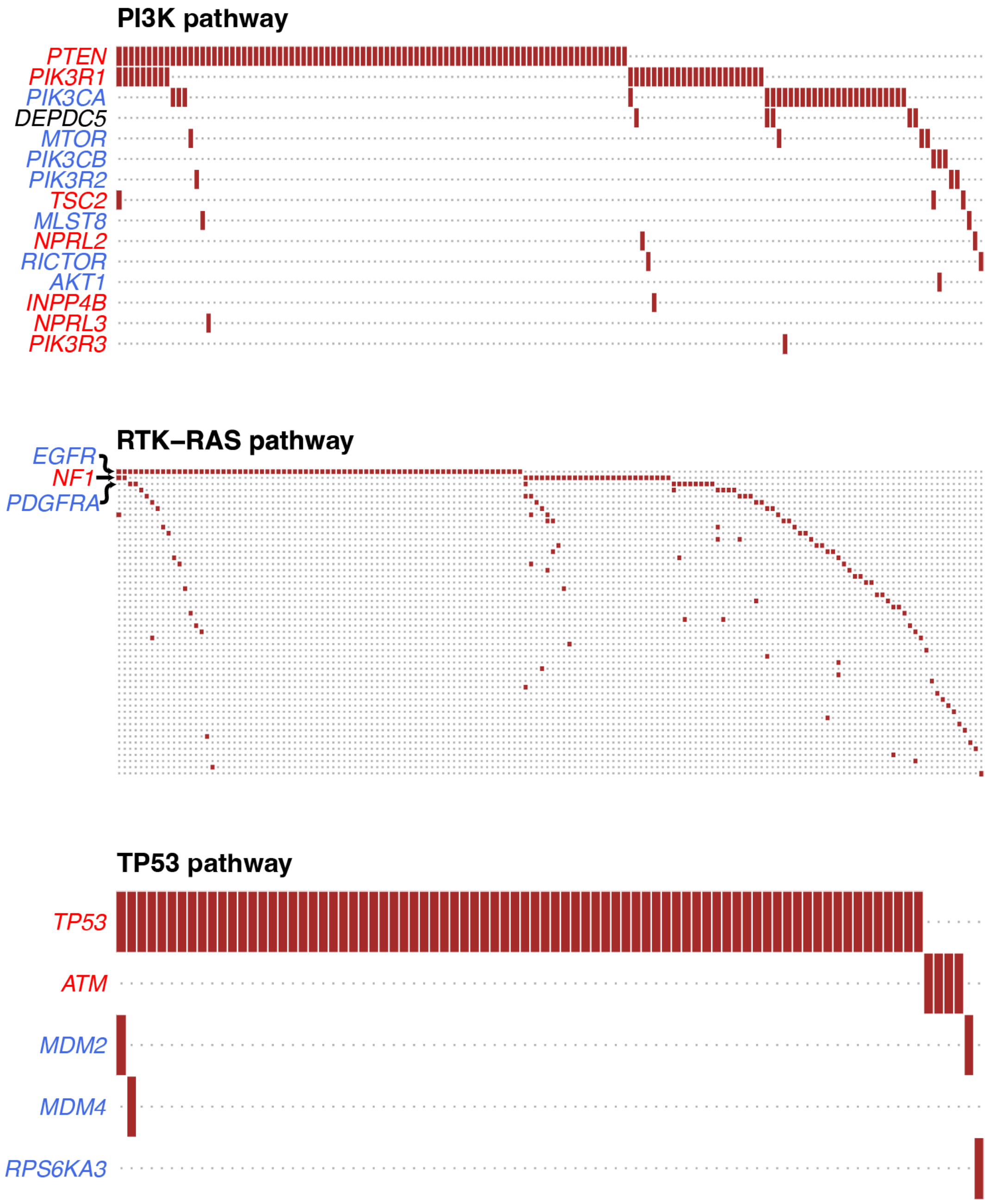

Supplementary Figure 3. Gene-level pathway analysis for highly mutated pathways. CRUX facilitates more granular investigation of individual pathways. Columns represent individual samples, with each coloured box representing a that the given gene is mutated in the sample. Gene names are coloured red for tumour suppressor genes and blue for oncogenes.

Sample Id	Gene	Protein change	Consequence	Domain	*Oncogenic classification*	GDNA
Search here...	TTN					
TCGA-DJ-A13O	TTN	p.L21223F	Missense	fn3	predicted passenger	chr2:g.179439488G>A
TCGA-DJ-A1QO	TTN	p.E7343D	Missense	I-set	predicted passenger	chr2:g.179580380C>G
TCGA-DJ-A2Q3	TTN	p.R27041*	Nonsense	fn3	predicted passenger	chr2:g.179419249G>A
TCGA-DJ-A3VA	TTN	p.P441Q	Missense	Titin_Z	predicted passenger	chr2:g.179659202G>T
TCGA-E3-A3E1	TTN	p.T18177Rfs*10	Frameshift	fn3	predicted passenger	chr2:g.179454219_17...
TCGA-E8-A417	TTN	p.V13170L	Missense	fn3	predicted passenger	chr2:g.179482973C>G
TCGA-EL-A3CU	TTN	p.T22056I	Missense	fn3	predicted passenger	chr2:g.179436988G>A
TCGA-EL-A3MY	TTN	p.Y30639C	Missense		predicted passenger	chr2:g.179402314T>C
TCGA-EM-A3AR	TTN	p.I28777M	Missense	fn3	predicted passenger	chr2:g.179412318T>C
TCGA-ET-A25K	TTN	p.K17826Q	Missense	fn3	predicted passenger	chr2:g.179455272T>G
TCGA-IM-A3U3	TTN	p.P3924T	Missense	I-set	predicted passenger	chr2:g.179598614G>T
TCGA-EL-A4K6	TTN	p.R14198*	Nonsense	fn3	predicted passenger	chr2:g.179476842G>A
TCGA-EM-A3ST	TTN	p.E20463*	Nonsense	I-set	predicted passenger	chr2:g.179441971C>A
TCGA-EL-A4KI	TTN	p.R3257C	Missense	I-set	predicted passenger	chr2:g.179629473G>A
TCGA-EL-A3ZS	TTN	p.S19050F	Missense	fn3	predicted passenger	chr2:g.179449515G>A
TCGA-EL-A3ZS	TTN	p.P24898H	Missense		predicted passenger	chr2:g.179428462G>T
TCGA-EL-A3TB	TTN	p.M31494T	Missense	Pkinase	predicted passenger	chr2:g.179399157A>G
TCGA-E8-A432	TTN	p.W31431R	Missense	Pkinase	predicted passenger	chr2:g.179399347A>T
TCGA-BJ-A0ZB	TTN	p.Y29510Y	Synonymous	I-set	not protein-affecting	chr2:g.179408637G>A
TCGA-DE-A4MC	TTN	.	IntronicSNV		not protein-affecting	chr2:g.179610827C>T
TCGA-EL-A3H7	TTN	p.S7441S	Synonymous	I-set	not protein-affecting	chr2:g.179579858G>A
TCGA-EM-A4FN	TTN	.	IntronicSNV		not protein-affecting	chr2:g.179611913T>G
TCGA-ET-A3BW	TTN	.	IntronicSNV		not protein-affecting	chr2:g.179610555A>G
TCGA-EL-A3CS	TTN	.	IntronicSNV		not protein-affecting	chr2:g.179612467A>G

Supplementary Figure 4. Cancer Genome Interpreter (CGI) annotation of Titin (*TTN*) variants present in the TCGA thyroid cancer cohort. The most severe oncogenic classification of *TTN* variants is 'predicted passenger', which indicates *TTN* mutations are very unlikely to drive thyroid cancer development, so *TTN* cannot be classified as a tumour driver gene.
